# Differential Beta-Band Event-Related Desynchronization during Categorical Action Sequence Planning

**DOI:** 10.1371/journal.pone.0059544

**Published:** 2013-03-18

**Authors:** Hame Park, June Sic Kim, Chun Kee Chung

**Affiliations:** 1 Department of Neurosurgery, MEG Center, Seoul National University Hospital, Seoul, Korea; 2 Department of Neurosurgery, Seoul National University Hospital, Seoul, Korea; 3 Department of Neurosurgery, Seoul National University College of Medicine, Seoul, Korea; 4 Sensory Organ Research Institute, Seoul National University, Seoul, Korea; University of Reading, United Kingdom

## Abstract

A primate study reported the existence of neurons from the dorso-lateral prefrontal cortex which fired prior to executing categorical action sequences. The authors suggested these activities may represent abstract level information. Here, we aimed to find the neurophysiological representation of planning categorical action sequences at the population level in healthy humans. Previous human studies have shown beta-band event-related desynchronization (ERD) during action planning in humans. Some of these studies showed different levels of ERD according to different types of action preparation. Especially, the literature suggests that variations in cognitive factors rather than physical factors (force, direction, etc) modulate the level of beta-ERD. We hypothesized that the level of beta-band power will differ according to planning of different categorical sequences. We measured magnetoencephalography (MEG) from 22 subjects performing 11 four-sequence actions - each consisting of one or two of three simple actions - in 3 categories; ‘Paired (ooxx)’, ‘Alternative (oxox)’ and ‘Repetitive (oooo)’ (‘o’ and ‘x’ each denoting one of three simple actions). Time-frequency representations were calculated for each category during the planning period, and the corresponding beta-power time-courses were compared. We found beta-ERD during the planning period for all subjects, mostly in the contralateral fronto-parietal areas shortly after visual cue onset. Power increase (transient rebound) followed ERD in 20 out of 22 subjects. Amplitudes differed among categories in 20 subjects for both ERD and transient rebound. In 18 out of 20 subjects ‘Repetitive’ category showed the largest ERD and rebound. The current result suggests that beta-ERD in the contralateral frontal/motor/parietal areas during planning is differentiated by the category of action sequences.

## Introduction

### 1. Activity Before Action

The word ‘Animal’ in Korean and Japanese is expressed by a set of two Chinese characters (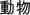), which means “moving object”. Moreover, humans are capable of highly sophisticated reasoning or thinking. Hence we plan before taking actions, making inferences about the consequences of our movements. Since the first measurement of the readiness potential [1], the physiological representation of the neural activity *prior* to motor execution has interested many researchers. Tanji and colleagues have been investigating the role of the prefrontal and non-primary motor areas in motor planning [2]–[5]. These papers showed that non-primary cortices and prefrontal cortex in monkeys have important roles in pre-movement planning. Subsequent studies focused on the non-primary motor cortices, and implemented tasks which involved cognitive aspects to planning movements [3], [6].

### 2. Planning Categorical Movement Sequences

A study [7] reported the existence of neurons in the dorso-lateral prefrontal cortex (DLPFC) of macaque monkeys, which fire while planning to execute upcoming motor sequence, selective of its action sequence category, rather than the individual sequences. We focused on searching how and where these neural activities representing abstract categorical information are manifested in the human brain at the whole-head range with non-invasive techniques. Although it is indicated in many primate studies that specific prefrontal and parietal areas are involved in a range of motor planning activities [8], the regions investigated in single-cell studies are limited. The paradigm used in Shima’s [7] study had a visual cue, auditory cue, and an associative memory component, and the anatomical areas involved in these functions should be spread throughout the brain. Therefore neural activities related to planning categorical action sequences may not be limited to a region in the prefrontal area - about 1 cm^2^, and may also take place in broader areas, especially the contralateral frontal/premotor/motor/parietal areas.

### 3. Beta-band Event-related Desynchronization (ERD) before Movement Onset

Numerous researchers strived to find the neurophysiological representation of the modulators for planning movements in human. The most prominent and reproducible observation is the beta power ERD in the motor related areas during movement preparation [9]–[11]. Beta-ERD has been reported to be a robust phenomenon which occurs approximately 2.0 ∼ 1.0 s prior to movement onset, of a pre-cued movement task [11]–[15] or a self-paced movement [16]–[21]. And as for what factors modulate the beta-ERD, studies have shown that movement direction, force, velocity [22], [23] or interception timing in a catching task [22], [24] or tiredness of the performing hand [25] did not have significant effect on the amplitude or magnitude of the beta-band ERD. However, Tzagarakis et al. [15] reported that the amount of beta-ERD varied as a function of response uncertainty; the larger the uncertainty for movement direction, the smaller the amount of beta-ERD. Another study [26] showed that anticipation of upcoming motor demand (movement inhibition prior to unloading weight) produced pre-movement beta-ERD, whereas unexpected unloading of weight did not. Therefore, we focused on the beta-band power time-course to see whether the amount of beta-ERD could be a discriminator among the categories of the upcoming action. We hypothesized that planning for different categorical action sequences would induce different levels of beta-ERD in the contralateral frontal/premotor/parietal areas.

## Materials and Methods

### 1. Ethics Statement

Prior to the study, all subjects submitted written informed consent for participating in the study. This study was approved by the Institutional Review Board of the Seoul National University Hospital (H-1102-022-350).

### 2. Subjects

24 right-handed healthy volunteers (10 females, age range = 21 ∼ 37 yrs, mean = 28.0 yrs, SD = 4.6 yrs) participated in the study. Two subjects (both males) were excluded (one subject’s performance was not recorded due to technical malfunction, and the other subject was not able to follow the instructions during measurement). The remaining 22 subjects’ data were included in the study. All subjects had normal or corrected-to-normal vision with no reported history of neurological or psychiatric illness.

### 3. Experiment Setup

The subjects were seated in a chair inside a magnetically shielded room facing a semi-transparent screen located 1.5 m from the subject’s eyes. A table was fixed to the chair upon which the subjects relaxed both arms. Visual cues were back-projected from a projector located outside the shield room onto the screen. The paradigm sequence was designed by STIM2 (Neuroscan, El Paso, TX, USA). The visual angle of the height of the visual cue circle was 2.7°. The subject’s head was placed inside the helmet and a soft cushion was placed behind the head when necessary in order to keep the subject’s forehead on the frontal part of the helmet.

### 4. Task

**Figure 1:**
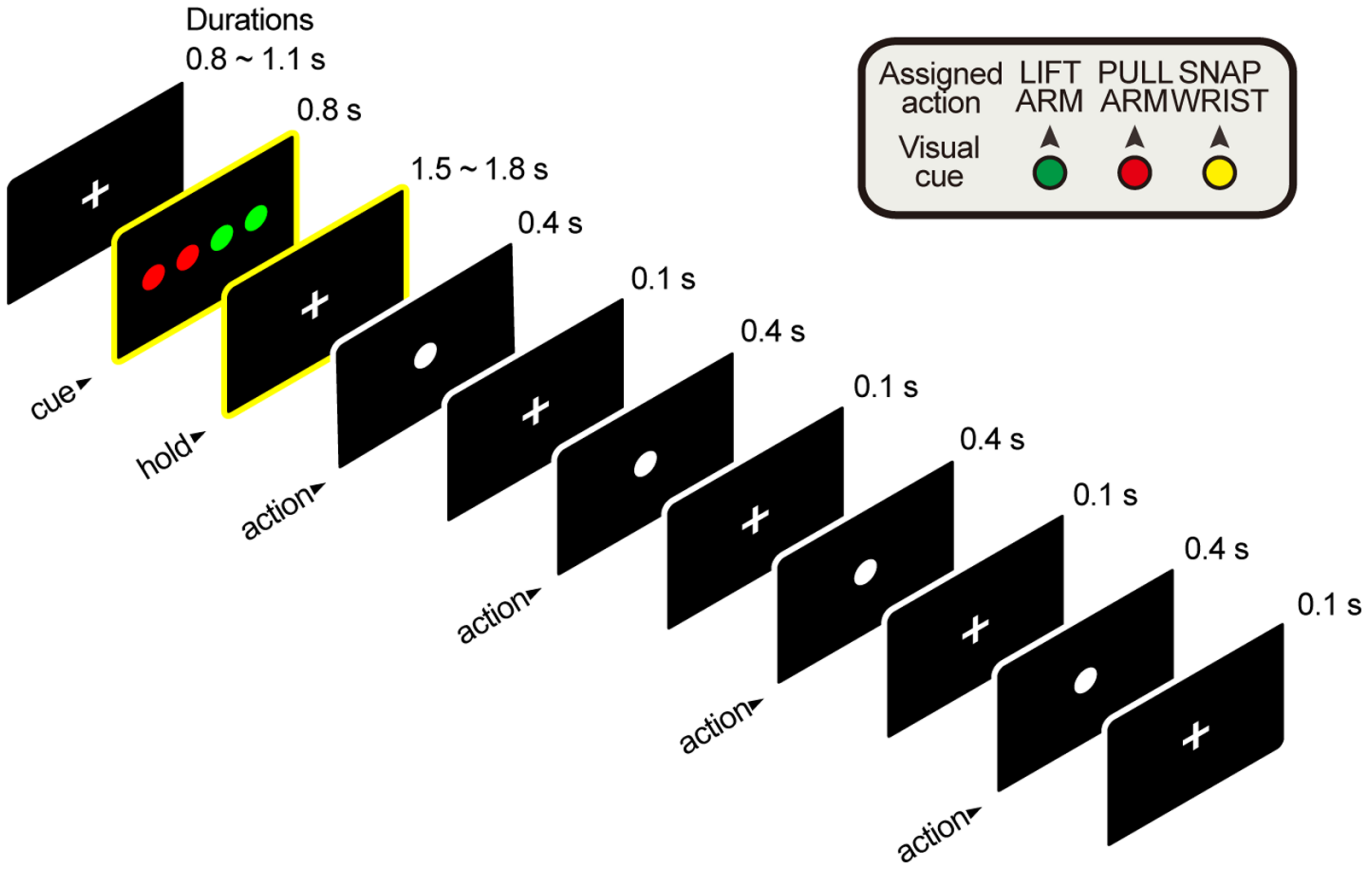
Schematic drawing of a 'Paired' trial. The frames highlighted with yellow borders are the phase of interest. Data obtained during this period were analyzed.

Subjects engaged in a visual-cue-instructed delay-and-action task, which was adapted from Shima et al. [7]. The subjects were to perform a four-component action sequence each comprised of one or two of three simple movements with the right hand or arm. From the resting position, which was to relax both arms on the table, lifting the right arm 45° from the table with the elbow acting as a fulcrum, was assigned to color green. Shifting the right arm inwards 45° to the torso with the elbow acting as an axis was assigned to color red. Dorsiflexion of the right hand 45° from the wrist joint was assigned to color yellow. These movements were selected as the three simplest and easiest movements a subject can perform with the arm or hand excluding fingers within the experiment environment. A ‘Paired’ sequence was composed of either green + red, or red + yellow actions in 2 paired sequences (e.g. red-red-green-green). An ‘Alternative’ sequence was composed of either, green + red, or red + yellow actions in 2 alternative sequences (e.g. red-green-red-green). A ‘Repetitive’ sequence was composed of either green or red or yellow actions in 4 repetitive sequences (e.g. red-red-red-red). There were a total of four kinds of sequences for ‘Paired’ and ‘Alternative’ and three kinds for ‘Repetitive’. A total of 275 trials were presented (100 trials for ‘Paired’ & ‘Alternative’ sequences, 75 for ‘Repetitive’ sequences) pseudo-randomly. A trial started when the subject fixated on the fixation point on the screen. Four colored circles were presented after the fixation period. The action was initiated with a go signal (first white circle) after a delay period of 1.5 ∼ 1.8 s from the visual cue offset. A schematic image of the experiment is presented in Figure 1.

Since the original task was designed for macaque monkeys and for extra-cellular recordings, significant adjustment on the details was made in order to fit the cortical magnetic fields measurement protocol for human subjects. Two of the most prominent differences were; first, whereas the original task was to perform a memory-based action sequence after five [7] visually guided trials, the current design did not include guided trials and proceeded directly to memory-based trials throughout the whole session. Subjects learned the rules for the task outside the shielded room prior to starting the session. The subject entered the shield room when they were fully capable of performing the correct actions assigned to each color coded circles. Second, whereas in the original task the colored cues were presented by LEDs (light emitting diodes) in serial, in the current design, the four cues were presented in a single frame, which significantly reduced the length of the session, thus enabling us to acquire the number of trials required for time-locked averaging of the data. Also the reduction helped to maintain alertness throughout the session which was necessary in order to obtain favorable performance.

### 5. Magnetoencephalography/Electrophysiological Signal Measurements

Magnetic signals were recorded by a 306 channel whole-head Elekta Neuromag Vectorview system (Elekta Neuromag Oy, Helsinki, Finland). The system has two planar gradiometers and one magnetometer as a single sensor unit, distributed at 102 locations forming a helmet shape. Signals were analogue filtered at 0.1 ∼ 200 Hz, sampled at 600.615 Hz and stored offline for further analyses. Four head position indicator (HPI) coils were attached to the subject’s head. The locations of the HPI coils with respect to three anatomical landmarks; the nasion, and two bilateral pre-auricular points, were digitized with a 3-dimension digitizer (Fastrak, Polhemus, Burlington, VT, USA). Electrooculograms (EOG) and electrocardiograms (ECG) were obtained simultaneously at the same sampling rate as the magnetic fields, in order to detect artifacts from the eyes and heart beat. Also surface electromyogram (EMG) was obtained via an electrode placed on the skin covering the extensor carpi ulnaris of the right arm in order to detect movement onset and additional artifacts. An MEG compatible camcorder was fixed on a table beside the subject and recorded the subject’s movement during the session in order to check for abnormal activities and performance accuracy.

### 6. Magnetoencephalography Preprocessing

All raw MEG data were preprocessed with the temporal signal space separation algorithm (tSSS) Maxfilter 2.2.10 (Elekta Neuromag Oy, Helsinki, Finland) [27], [28] in order to eliminate environmental and movement noise. Remaining analyses were performed with MATLAB 7.5.0.342 (MathWorks Inc., Natick, MA, USA), a toolbox FiffAccess 1.2 (Brain Research Unit, Low Temperature Laboratory, Helsinki University of Technology, Helsinki, Finland) and custom-built in-house codes. Independent component analysis [29] was applied to signals from frontal lobe sensors in order to extract EOG artifacts, and then reconstructed for further analysis. Additional artifacts were inspected manually. The events for all visual cue onset time points were extracted and overlayed with EMG signals in order to search for movement artifacts, and also the recorded videos were inspected in order to check for movement artifacts and performance errors. Only the correct trials were included in the analyses. Then the events were grouped by the three categories and further data were processed separately based on these events for each category.

### 7. Time-Frequency Representation (Event-Related Spectral Perturbation)

A Morlet wavelet transform algorithm was used to calculate the time-frequency representation of the neural activity. A wavelet defined by
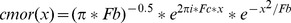
where *Fb* is the bandwidth parameter and *Fc* is a wavelet center frequency. Here, *Fb = *2.48 and *Fc = *1. The wavelet had approximately 6 cycles, providing high temporal resolution at high frequencies with low spectral resolution, and vice versa at the low frequencies. Therefore this was an optimized parameter for estimating the beta-band power. The time window of an epoch was defined between – 0.2 ∼ 2.3 s where zero was the visual cue onset. This time window covered the visual cue display duration plus the waiting period (1.5 ∼ 1.8 s after visual cue offset) before movement cue onset. Wavelet transform coefficients were calculated for the full-length data of a session for all gradiometer channels (only the gradiometer data were used in the analyses). The absolute value of the coefficients was epoched for each category, and then averaged. Baseline was from - 0.2 ∼ 0.0 s. Power was calculated as:



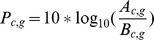
where *c* is for all three categories, *g* is for all gradiometer sensors, *P* is power in decibels, *A* is averaged wavelet transform coefficients for the full epoch (- 0.2 ∼ 2.3 s), and *B* is the averaged wavelet transformation coefficients at baseline. We then normalized the power with the level of power during 0 s ∼ 0.2 s after the first movement onset (P_mov_) determined by the EMG data for all trials as follows [15]:



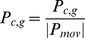



The power level at the movement onset converged to a similar level for all categories. The resulting values were from -1 to 0, for most subjects, where -1 indicated the power value upon movement onset.

### 8. Beta-band ERD and Transient Rebound

**Figure 2:**
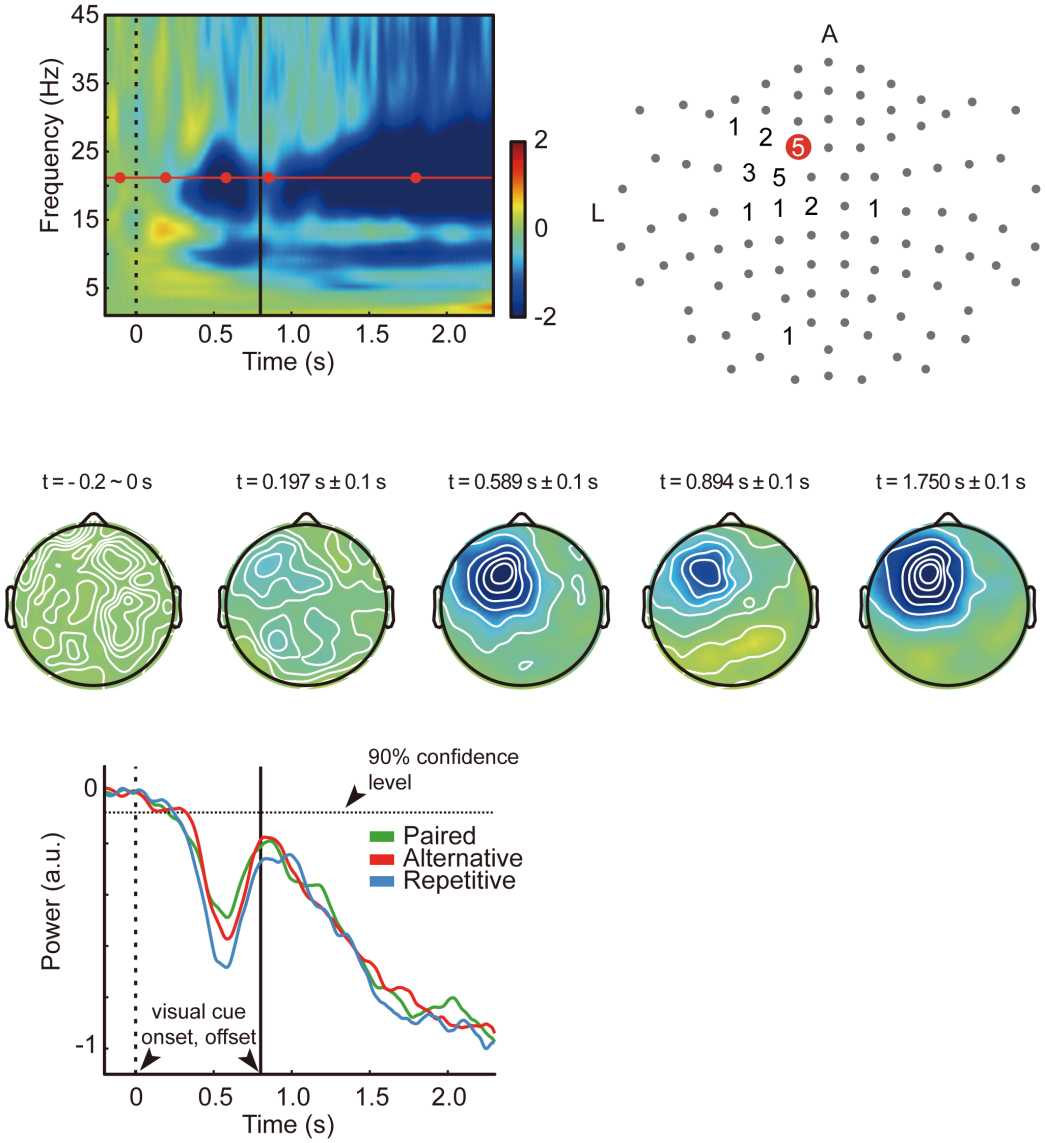
Beta power results from a representative subject. *Top row;* Time-frequency representation of data from the red sensor on the right plot for Subject 20. The horizontal red line is 21 Hz, the frequency with maximum beta power difference during visual cue display. Color bar is power in arbitrary units. The dotted and solid vertical lines are zero (visual cue onset) and visual cue offset (0.8 s), respectively (left). 102 channel full view plot and the number of subjects located at channels with the maximum beta-band power difference (right). Grey dots depict channel locations. *Middle row;* Topology plots of beta power during motor planning. The time points correspond to the red dots on the time-frequency plot above. Color coding is same as the time-frequency plot. *Bottom row;* 21 Hz power overlayed for three categories.

**Figure 3:**
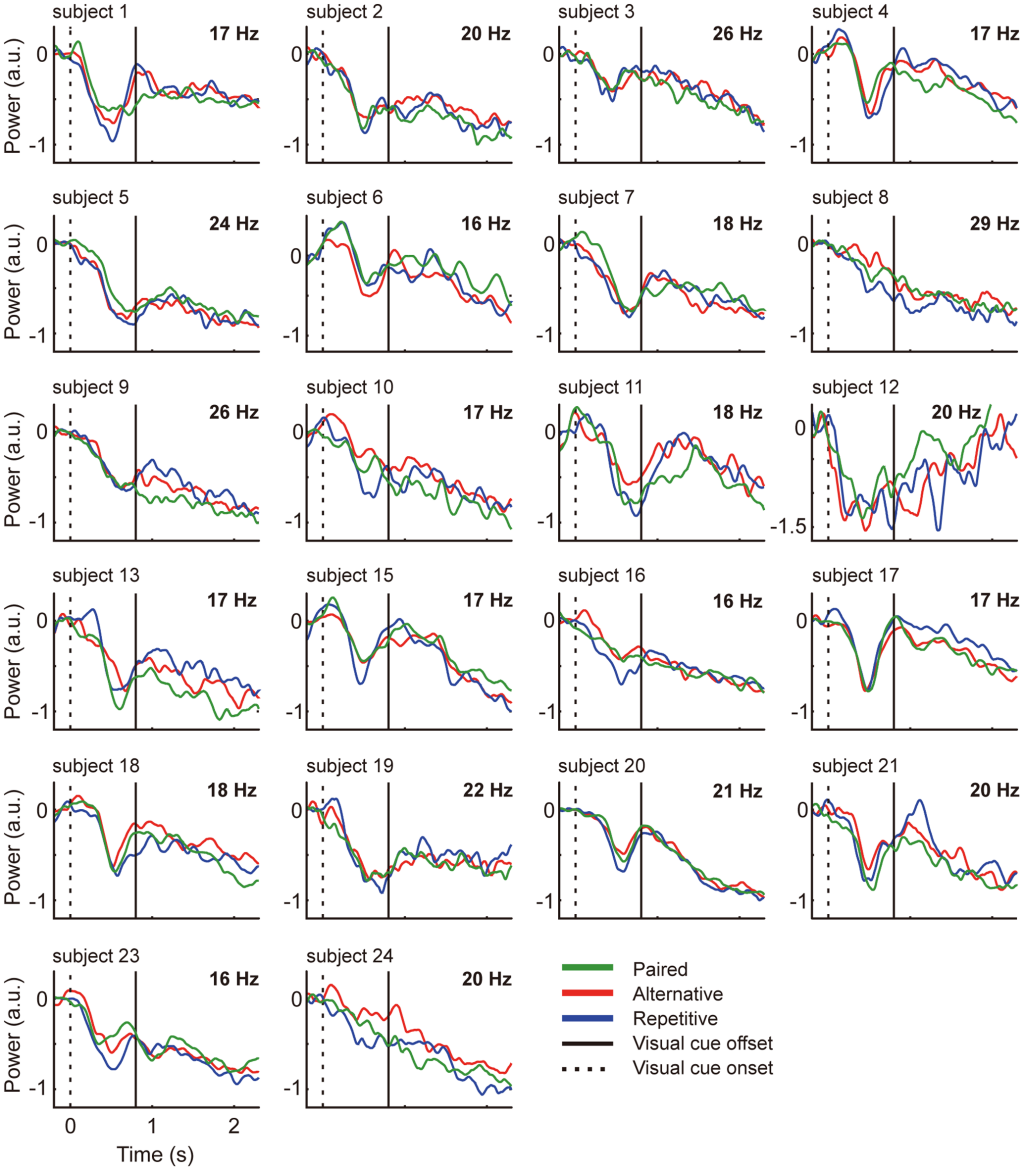
Beta-power time-course for 22 subjects. Vertical axis is power (arbitrary units), horizontal axis is time (second). Green line is ‘Paired’ red line is ‘Alternative’ and blue line is ‘Repetitive’. The dotted and solid vertical lines depict zero (visual cue onset) and visual cue offset (0.8 s), respectively. The frequency of the power for each subject is indicated in the upper right corner of each plot.

Since the subjects’ head size and relative position regarding the fixed sensor location varied, the location for the most prominent beta-ERD differed across subjects. Therefore, we decided that selecting a common single channel for all subjects may not provide the accurate information for the cortical activity underlying the neural events taking place during motor planning. Also, averaging across channels with clustering methods may result in different numbers of channels for each subject, which will result in different sensitivities for statistical analyses. Instead, we found for each subject the channel and frequency with the most prominent beta-ERD by calculating the maximum ERD value within the beta-band (15 ∼ 30 Hz) during visual cue display (0.0 ∼ 0.8 s), from beta-band power time-course averaged by all trials. More specifically, this was calculated as the difference between the maximum value prior to power decrease and the minimum value during visual cue display. The reason we have chosen a peak-to-peak measure for comparing power levels for the three conditions is that the latencies of peaks of three conditions were not time-locked. What we were interested in was the difference of beta power among the three categories during motor planning. The selected channels for all subjects were mostly confined within the area covering the contralateral frontal and anterior parietal areas, although two subjects had the most prominent beta-ERD in sensors located in the contralateral posterior parietal area and ipsilateral posterior area. The locations of selected channels for all subjects are displayed in Figure 2. The frequency for all subjects was within the range of 16 ∼ 29 Hz (mean = 19.64 Hz, SD = 3.68 Hz). The frequency for each subject is indicated on the upper right corner of each plot in Figure 3. Likewise, the transient rebound was defined as the difference between the minimum value during visual cue display, and the maximum value after the time point of the beta ERD minimum. For subject 12, since the beta-band time-course did not show a peak after ERD, we could not define a peak rebound value and the data were not included in statistical analysis. The onset of beta-ERD was calculated as the time point when the beta power decreased below the lower value of the 90 % confidence interval for - 0.15 ∼ 0.05 s (during the baseline period) [15].

### 9. Statistical Analysis

We used a linear mixed model (LMM) [15], [30] to test for statistical differences between power values across action sequence categories. Since the experimental design had multiple conditions within a single subject, correlation within subject data had to be accounted for, and since the beta-ERD and transient rebound values had large variance between subjects, we accommodated the mixed model in order to consider these factors when comparing relative power values across categories across subjects. A variance components structure was used as a covariance structure, with action category as the fixed effect and subjects as the random effect. The category-wise maximum values of beta-ERD and transient rebound amplitudes were the dependent variables respectively. All datasets were tested and confirmed graphically for normality in advance [31], [32]. Results at p < 0.05 were considered to be statistically significant for all tests. Statistical tests were performed with MATLAB and IBM SPSS® Statistics 17.0.0 (SPSS Inc., IBM Company, Chicago, IL, USA).

### 10. Beta-Band ERD Source Re-construction

In order to reconstruct the sources for beta-ERD, we used a modified linearly constrained minimum variance beamformer [33] implemented in BESA 5.1.4 (MEGIS Software, Gräfelfing, Germany). The multiple source beamformer which performs source imaging of EEG/MEG data in the time-frequency domain filters the activity for each voxel grid in the brain volume with the activities from all magnetic field signals recorded from all sensors. Therefore, beamformers provide the estimate of the contribution of a single brain position to the measured field.

The data used were as follows; the activity during maximum ERD contrasted with the same length of data during baseline period prior to visual cue onset was used. These time windows varied across subjects since the duration of desynchronization was different for each subject. The average time range (same length for baseline) for ERD was 338.10 ms (SD = 65.01 ms). The difference of source activity compared to the activity during baseline is plotted in percentage. The source activity was calculated and exported to Brainvoyager QX 2.3 (Brain Innovation) from BESA. The source activity was overlayed on a BESA default MNI template. These volume maps were then imported to Brainvoyager QX and averaged to create a grand average of the source activity.

## Results

### 1. Behavioral Results

Mean error rate for all trials across subjects was 0.81 % (SD = 1.41 %); 0.86 % (SD = 1.24 %) for ‘Paired’, 0.74 % (SD = 1.31 %) for ‘Alternative’, and 0.83 % (SD = 1.32 %) for ‘Repetitive’ sequences. There was no difference in error rates across categories (F_(2,63)_ = 0.04, p = 0.96). more specifically, the errors were divided into two types; performance error and inhibition error. Performance error was when the subject did not perform the action sequences according to the visual cue presented. Inhibition error was when the subject initiated the first movement before movement cue onset. As for performance error rates across all subjects, ‘Paired’ was 0.31 % (SD = 0.64 %), ‘Alternative’ was 0.35 % (SD = 0.85 %) and ‘Repetitive’ was 0.13 % (SD = 0.26 %). Inhibition error rates were 0.55 % (0.76 %) for ‘Paired’, 0.40 % (SD = 0.87 %) for ‘Alternative’ and 0.69 % (SD = 1.34 %) for ‘Repetitive’. Although subjects tended to make more inhibition errors for ‘Repetitive’ (84 % of all ‘Repetitive’ errors) than the other two (63 % and 53 % for all of ‘Paired’ and ‘Alternative’ errors, respectively), a paired-sample T-test did not confirm that the error rates for any of the error types were significantly different among categories (p > 0.05).

### 2. Time-frequency Representation

As we have expected, from the time-frequency representation plots for all subjects, beta-ERD after visual cue onset was clearly visible for all categories. For most subjects, the most prominent beta-ERD was localized around the contralateral frontal/anterior parietal areas (Figure 2). One subject showed prominent beta-ERD in the posterior parietal area and another subject showed the strongest beta-ERD mostly in the ipsilateral frontal/parietal area.

Mean latency for maximum beta-ERD during visual cue display was 542 ms (SD = 75 ms) for ‘Paired’, 571 ms (SD = 109 ms) for ‘Alternative’ and 572 ms (SD = 97 ms) for ‘Repetitive’. The values were not significantly different from each other (p > 0.05).

The mean latency for maximum transient rebound was 965 ms (SD = 252 ms) for ‘Paired’, 974 ms (SD = 276 ms) for ‘Alternative’ and 998 ms (SD = 204 ms) for ‘Repetitive’. The values were not significantly different from each other as well (p > 0.05).

The average beta-ERD onset latencies for 21 subjects were 259 ms (SD = 88 ms) for ‘Paired’, 222 ms (SD = 98 ms) for ‘Alternative’, and 217 ms (SD = 111 ms) for ‘Repetitive’. The onset latencies were not different over categories (p > 0.05).

### 3. Beta-ERD, Transient Rebound

**Figure 4:**
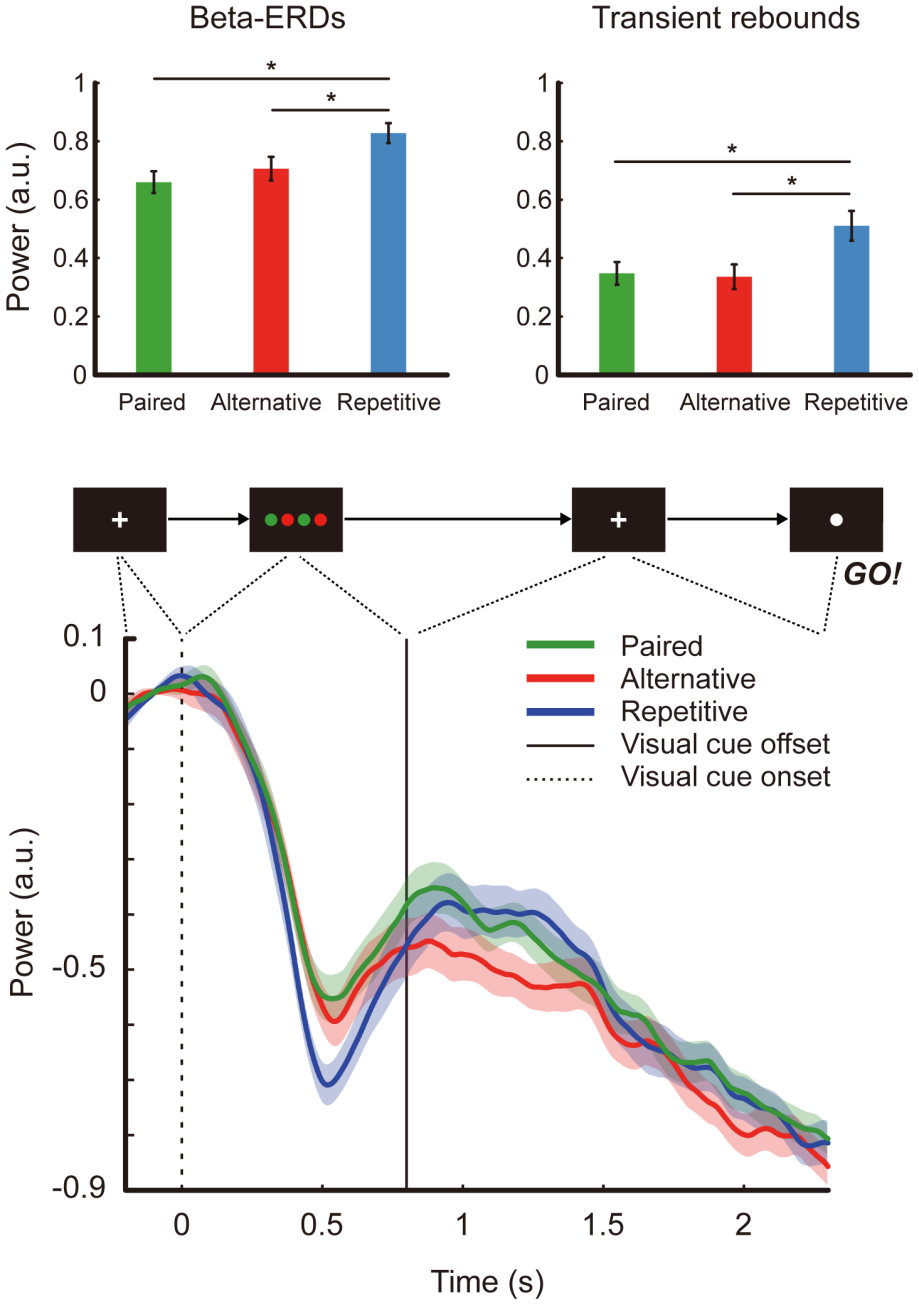
Result summary. Power differences for beta-ERD during visual cue display (upper left), and transient rebound following maximum ERD (upper right), and the averaged beta power time-courses for 21 subjects (bottom). The vertical axis is power in arbitrary units. Green line is ‘Paired’ red line is ‘Alternative’ and blue line is ‘Repetitive’. Asterisks show statistical significance at p < 0.05 by the linear mixed model. Error bars indicate standard errors.

The mean and standard deviation for the beta-ERDs in arbitrary units were 0.66 (SD = 0.17) for ‘Paired’, 0.71 (SD = 0.04) for ‘Alternative’ and 0.83 (SD = 0.16) for ‘Repetitive’. These values were confirmed to be statistically different among categories by a univariate test on the effect of categories based on the linearly independent pair-wise comparisons among the estimated marginal means from the linear mixed model (F_(2,40)_ = 22.27, p < 0.0001). Between categories, beta-ERD during visual cue display was found to be significantly different between ‘Repetitive’ and ‘Alternative’ (p < 0.0001), and ‘Repetitive’ and ‘Paired’ (p < 0.0001), but not between ‘Alternative’ and ‘Paired’ (p > 0.05). Likewise, the mean and standard deviation for the transient rebounds were 0.35 (SD = 0.18) for ‘Paired’, 0.34 (SD = 0.19) for ‘Alternative’, and 0.51 (SD = 0.22) for ‘Repetitive’. These values were also statistically different among categories (F_(2,40)_ = 20.42, p < 0.0001). Also between categories, transient rebounds were found to be significantly different between ‘Repetitive’ and ‘Alternative’ (p < 0.0001), Repetitive’ and ‘Paired’ (p < 0.0001), but not between ‘Alternative’ and ‘Paired’ (p > 0.05). Significance levels were adjusted for multiple comparisons with Bonferroni correction. These results are summarized in Figure 4. Beta-power time-course plots for all 22 subjects can be seen in Figure 3. Figure 4 shows the grand average aligned to visual cue onset for 21 subjects (bottom plot).

### 4. Regions of Beta-ERD

**Figure 5:**
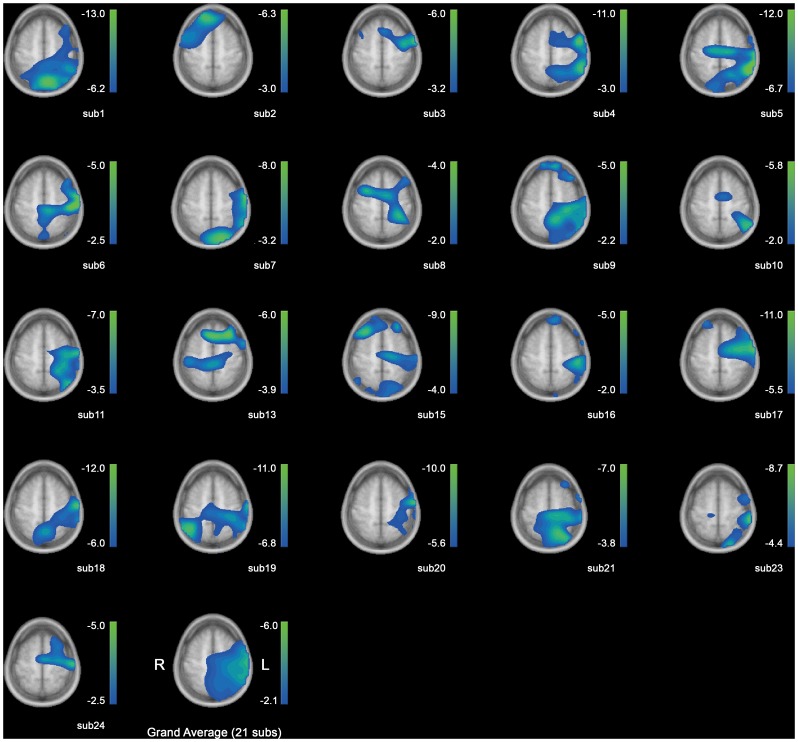
Source reconstruction for beta-ERD for 21 subjects. From the upper left to right; subject 1 ∼ 5, in ascending order down to subject 24 in the bottom line, excluding subjects 12, 14 and 22. Grand average is on the last grid. The numbers in the scale bars indicate the amount of activity change from baseline in percent. Left is right and right is left.

The source activities for the beta-ERD are shown in Figure 5. The percentage of change from baseline is indicated in the scale bars next to each head model. The activities are overlayed on the same slice for all subjects (z = 75 mm), which reveals the hand knobs on motor areas. From Figure 5, the activity is consistently located in the contralateral hemisphere of the performing arm, except for Subject 2. The area covers a wide range from the prefrontal/premotor cortex to supplementary motor area in some subjects, and to the posterior parietal cortex in most subjects. The averaged image (Figure 5, last grid) shows a common region of activity corresponding to these areas as well. The origins of the beta-ERDs computed from the signals at the sensor level are thus speculated to lie in the contralateral motor and associative areas.

## Discussion

### 1. Differential Beta-ERDs in Motor Planning

We have found differential beta power time-course during categorical action sequence planning in human beings. The differences in beta power amplitude occurred during visual cue display and in a transient rebound following the maximum beta-ERD. Previous studies reported beta-ERDs induced by visual cues [34], [35] during visual cue display. Especially, Müller-Gerking et al. [34] were able to discriminate the upcoming limb movement (right or left finger or foot) from the ERD signals during visual cue display. They suggested that not only the planning or imagination of a movement, but also visual cues indicating a certain limb movement can induce somatotopically specific beta-band ERD. Since our paradigm utilized a single limb for all movements, and only the components of movement differed, adopting the results directly from these reports to the current finding may not be valid. However, with a similar associative memory based delayed task paradigm, it is notable that in our study the amount of beta-ERD reflecting preparation for activating a single limb was differentiated according to upcoming categorical action sequences.

Meanwhile, previous studies on processing visual signals for movement tasks [36], [37], have shown that neurons in the premotor area process visual information which guides future movements. These visual cues may be direct instructions of limb movements, or indirect specifications of conceptual actions. Especially for visual cues which are conceptual representation of action targets as in our study, neurons in the dorsal premotor area are reported to be involved in the process. In similar context, the premotor cortex in primates have been suggested to convert an abstract concept for action into a concrete motor plan [38]. In accordance with these previous studies, in our results, the source of beta-ERD reconstructed for the time window of maximum ERD during visual cue display (Figure 5) encompasses a wide region in the contralateral hemisphere, including the premotor area.

Meanwhile, in a previous MEG study, the amount of beta-band activity during motor planning was related to the degree of uncertainty of the upcoming response [15]. More specifically, the number of cues provided as the first cue modulated the level of beta-ERD in a negative correlation. The level of uncertainty may be considered as the level of task load (being ready for any direction vs. knowing which direction to proceed). Being certain of the direction to move led to a greater beta-ERD after cue onset. This leads to the speculation that the categorical action sequences used in the current study may have different preparation loads. And the different preparations may be due to movement complexity of sequences [39], [40]. However, in this study, the overall error rates among the three categories did not show statistical difference, and the movement onset latency among the three categories did not differ significantly either. In a study on the elements which affect the reaction time (RT, movement onset latency) in a pre-cued movement task [41], [42], the authors found that in a condition where the subjects knew what action to execute prior to the go cue (as in our paradigm), the reaction time increased as the task became complex. The task from this study also had a delay period before the go cue, allowing the subject to have time to plan before execution, as in our study design. Therefore, if the ‘Paired’ and ‘Alternative’ conditions were more ‘complex’ than the ‘Repetitive’ condition in our paradigm, the difference in reaction time should have been observed in this study accordingly, which was not. Overall, it may be premature to conclude that the differing levels of complexity between the three categorical movements may have been the reason for the differentiated amplitudes during motor planning. It should be noted, however, that we cannot conclude that the complexity effects were completely absent from this paradigm only. Further study with complexity as a parameter for motor planning would reveal how beta-ERD is modulated by the complexity of sequential movements.

Another point which must be taken into consideration is, unlike previous studies which used gradable stimuli (i.e., degree of uncertainty, degree of force etc.), in our study, the visual cues are not gradable, or rather neutral. It is possible then that the three categories had different effects on the level of beta-ERD within subjects. Especially, since the ‘Paired’ and ‘Alternative’ categories have similar construct in sequence and in visual combination (i.e., two colors), when categorizing the three patterns (ooxx, oxox, oooo), the order may have been different for the two more similar cues, according to each subject. This effect may have been smeared when averaged across all subjects.

### 2. Transient Rebound after Maximum ERD

Following the beta-ERD, subsequent transient rebound was also detected in almost all subjects, starting while visual cue display and mostly peaking after visual cue offset. There have been reports concerning brief beta-rebounds after termination of motor imagery of foot or hand in the frontal, central and parietal areas [43]–[45]. Notably, the beta-rebound was prominent over the contralateral side of the head after imagery of the hands. From our result, the transient rebound can be regarded as an event-related synchronization (ERS) *relative* to the beta-ERD during visual cue display. This may be a neural reflection of covert simulation for the planned action sequence, which is induced by the action-coded visual stimuli. Also beta-ERS has been constantly linked to action inhibition [35], [46], which is usually reported to occur after movement completion. However, intentional inhibition of planned actions has also been found to be related to beta-ERS when subjects did not choose to act following conscious intention to act [47]. We can speculate that in correct trials, the transient rebound may reflect successful intentional inhibition until the go cue was given.

### 3. Conclusion: Strength, Limitations, and Further Studies

The strength of the current study lies in that it was an attempt to find how the remarkable results from a macaque single-cell recording paradigm [7] may be studied at a large population level, with a whole-head non-invasive MEG study on humans. It showed that the beta-activity during motor planning is modulated by the category of the action sequence to perform. This is a new factor that may be able to modulate the level of beta-ERD, which is also a cognitive component, rather than a physical component, such as direction, force, and velocity as mentioned in 1.3. In addition to the beta-ERD which is a typical feature occurring between the first cue and a go cue in pre-cued motor planning paradigms, the subsequent transient rebound was also modulated by the type of action category to perform. These induced activities were most prominent over the motor areas, extending to the parietal areas, suggesting that planning for different types of action sequences may constitute a larger network than that implied by Shima et al.’s [7] study.

However, the beta-activities for ‘Paired’ and ‘Alternative’ sequences were not found to be significantly different at the group level in the current scope of analyses. Despite the discussion in 4.2, in order to further investigate the neural activities during motor planning of distinctive action sequences, the correlation between the visual-cue induced associative memory retrieval process and subsequent motor planning should be taken into account in a long distance networking level. This network may include the occipital-frontal connection, and the fronto-parietal connection for. Although the current range of analysis did not provide any network information between distinct areas within the motor planning network, in some subjects, prominent ERD/ERS activities in areas such as the occipital, prefrontal areas were observed (data not shown). The latencies of maximum ERD for both beta and alpha powers were in the order of occipital - frontal - parietal for those subjects. Although this is only an implication for an existing long-range network involving visual functions and cognitive functions such as associative memory retrieval, working memory during the delay period, and actual motor preparation, from the fact that activities are apparent in other frequencies as well as regions, measures such as the phase-amplitude modulation index [48] may provide interesting results, especially when the memory components can act as the crucial effective factor on the differential measure [49], [50]. However, the memory component was not the test variable in our study, i.e., the subjects practiced thoroughly outside of the shielded room, and since the movements and visual cues were both very simple, we did not expect the difference in working memory load would affect neither the performance nor the results. Further study is promising to reveal these unsolved issues in the field of motor planning in humans.
